# Co-exposure of potentially toxic elements in wheat grains reveals a probabilistic health risk in Southwestern Guizhou, China

**DOI:** 10.3389/fnut.2022.934919

**Published:** 2022-08-08

**Authors:** Dashuan Li, Cheng Zhang, Xiangxiang Li, Fuming Li, Shengmei Liao, Yifang Zhao, Zelan Wang, Dali Sun, Qinghai Zhang

**Affiliations:** Key Laboratory of Environmental Pollution Monitoring and Disease Control, Ministry of Education, School of Public Health, Guizhou Medical University, Guiyang, China

**Keywords:** potentially toxic elements (PTEs), source identification, non-carcinogenic risk, carcinogenic risk, probabilistic health risk, wheat grains

## Abstract

Bijie is located at a typical karst landform of Southwestern Guizhou, which presented high geological background values of potentially toxic elements (PTEs). Recently, whether PTE of wheat in Bijie is harmful to human health has aroused people’s concern. To this end, the objectives of this study are to determine the concentrations of PTE [chromium (Cr), nickel (Ni), arsenic (As), lead (Pb), cadmium (Cd), and fluorine (F)] in wheat grains, identify contaminant sources, and evaluate the probabilistic risks to human beings. A total of 149 wheat grain samples collected from Bijie in Guizhou were determined using the inductively coupled plasma mass spectrometer (ICP-MS) and fluoride-ion electrode methods. The mean concentrations of Cr, Ni, As, Cd, Pb, and F were 3.250, 0.684, 0.055, 0.149, 0.039, and 4.539 mg/kg, respectively. All investigated PTEs met the standard limits established by the Food and Agriculture Organization except for Cr. For the source identification, Cr and Pb should be originated from industry activities, while Ni, As, and Cd might come from mixed sources, and F was possibly put down to the high geological background value. The non-carcinogenic and carcinogenic health risks were evaluated by the probabilistic approach (Monte Carlo simulation). The mean hazard quotient (HQ) values in the three populations were lower than the safety limit (1.0) with the exception of As (children: 1.03E+00). However, the mean hazard index (HI) values were all higher than 1.0 and followed the order: children (2.57E+00) > adult females (1.29E+00) > adult males (1.12E+00). In addition, the mean carcinogenic risk (CR) values for Cr, As, Pb, and Cd in three populations were all higher than 1E-06, which cannot be negligible. The mean threshold CR (TCR) values were decreased in the order of children (1.32E-02) > adult females (6.61E-03) > adult males (5.81E-03), respectively, all at unacceptable risk levels. Moreover, sensitivity analysis identified concentration factor (C_*W*_) as the most crucial parameter that affects human health. These findings highlight that co-exposure of PTE in wheat grains revealed a probabilistic human health risk. Corresponding measures should be undertaken for controlling pollution sources and reducing the risks for the local populace.

## Introduction

Soil pollution by potentially toxic elements (PTEs) has spread worldwide, provoking the ecosystem and health risks to humans, owing to their stable, persistent, and irreversible properties (1). Crops grown in contaminated soils may accumulate PTE in their edible parts, resulting in an excessive human intake, which eventually poses adverse impacts to humans *via* the food chain (2–4). PTE can enter the human body in three ways including ingestion, inhalation, and dermal contact, while food consumption (>90%) has been recognized as the major pathway for human exposure (5). Therefore, from the perspective of both environmental security and human health, it is critical to pay more public attention to PTE contamination.

Being the most crucial toxic elements to humans, prolonged exposure to arsenic (As), chromium (Cr), cadmium (Cd), and lead (Pb) can make some threats even at low concentrations, such as kidney dysfunction, musculoskeletal systems, cholesterol balance, and central nervous system (6, 7). Nickel (Ni) is a carcinogenic element and its extraordinary amount can pose lung cancer, diabetes, and uremia (8, 9). Of note, although zinc (Zn) is beneficial to healthy human growth, it may defect in stomach cramps and reproduction (10, 11). According to the World Health Organization [World Health Organization (WHO) 2002 (12)], although fluorine (F) can help to strengthen bones and teeth (1–4 mg/kg normally), a daily intake (>6 mg/day) may be correlated with skeletal fluorosis, and a daily intake (>14 mg/day) may further cause serious risk of fracture (13). In recent decades, researchers have concentrated on the human health risk posed by PTE exposure in the food chain.

Wheat and wheat-derived foods play indispensable roles in human growth and have a critical position in food production, circulation, and ingestion (14, 15). China is one of the greatest wheat producers worldwide, accounting for 18% of the global wheat grain products (16). Durum wheat is consumed in a number of countries typically as pasta, noodles, and breakfast cereals; therefore, the demand for its production is increasing gradually (17). In terms of nutritional value, bran layers and embryo fractions of wheat produced by milling are abundant in minerals, fibers, and folate (18, 19). However, wheat can absorb some hazardous elements (e.g., Pb, Cd, and Ni), and the intake of excessive PTE through wheat and wheat-derived foods may pose health risks to humans (20). Research conducted in Baoji city showed a non-carcinogenic risk (CR) in wheat grains for children and adults due to Cr, Ni, Cu, Zn, Cd, and Pb exposure (21). Ali et al. (22) showed that the mean Pb concentration was above the permissible limit of the Food and Agriculture Organization (FAO), and its hazard quotient (HQ) was the highest (2.118) among different PTEs (e.g., Cr, Cd, As, and Zn). Nevertheless, few studies focused on wheat polluted by the co-exposure of F and other toxic elements so far.

To the best of our knowledge, two kinds of techniques including deterministic and probabilistic (Monte Carlo simulation) are well applied to estimate the human health risk due to several pollutants (23). The deterministic risk approach regarding PTE has been carried out in previous studies (24–26). Hu et al. (27) showed that HQ values for elements were significantly lower than 1 of the limit value using the deterministic without considering other variables, such as body weight (BW), ingestion rate (IR), and exposure durations (EDs), which eventually may underestimate the risk outcomes. Studies by Ihedioha et al. (24) and Guo et al. (28) were consistent with the study by Hu et al. Herein, the deterministic risk technique may exist some uncertainties during exposure assessment, leading to less persuasive risk results. In contrast, the probabilistic approach has attempted to emphasize the reliabilities and uncertainties of risk results based on the probability distributions of the PTE concentrations and related parameters (29). Actually, the probabilistic assessment in several studies has been applied. However, Jiang et al. (30) only considered for children to reduce the uncertainty of deterministic risk using the Monte Carlo simulation. Besides, Kukusamude et al. (5) performed the approach for exposure to elements in rice *via* consumption. Unfortunately, related exposure parameters (BW, IR, and ED) were not identified during probabilistic modeling (31). Based on the discussions earlier, the probabilistic assessment was applied in this study, considering essential parameters (C*, BW, ED, and IR), and the populations were divided into three groups based on age (children, adult females, and adult males), which will make the results more objective.

Southwestern China is the largest karst region worldwide and has been confirmed that the natural background of PTE was initially controlled by the lithology of parent rock and soil type-dependent (32). Various studies indicated that the maximum values of mean concentrations for PTE were distributed in karst regions such as Guizhou, Guangxi, and Yunnan provinces, which were attributed to unique geochemistry in the process of the soil formation (32, 33). As one of the most essential karst regions, Southwestern Guizhou has been reported that the background value for Cd (0.659 mg/kg) in soils was far higher than the standard value (0.097 mg/kg) in China, and the mean concentration for F was highly up to 1,200 mg/kg (34). Furthermore, the study region has been considered a typical fragile area for the well-developed industry (35). However, as one of the main cereals, any study is still not aware of the wheat pollution by PTE in this critical area.

In light of these facts, 149 wheat grains were collected and their PTE (Cr, Ni, As, Pb, Cd, and F) concentrations were measured by the inductively coupled plasma mass spectrometer (ICP-MS) and fluoride-ion electrode methods to (1) determine the concentration of PTE; (2) identify the pollution sources of PTE; and (3) assess the probabilistic risks (non-carcinogenic and carcinogenic) for children, adult females, and adult males. It will provide a new scope for pollution control and a scientific basis for local governance and will ensure the health of the populace.

## Materials and methods

### Study area and sample collection

**FIGURE 1:**
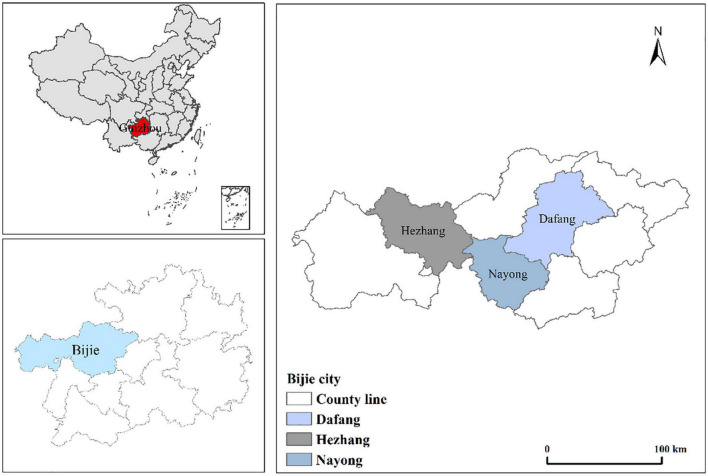
The map of the study area showing wheat grain sampling sites in Bijie, Guizhou Province.

Bijie (26°21′–27°47′N, 103°36′–106°44′E) is located in the Southwestern Guizhou Province, China. The total area of this region is 26,853 km^2^ and its plateau mountains account for 93.3%. It has a subtropical monsoon climate with an annual average temperature and precipitation, ranging from 12 to 15.4°C and 821 to 1,365 mm, respectively. The field survey has showed that it is not only a specific agricultural planting area, but its industry is well-developed in the surrounding area during the past decades. For instance, the area has vast mineral resource reserves ranking first in Guizhou Province, where the lead and phosphate reserves are up to 11 million tons and 1.41 billion tons, respectively. Meanwhile, it is considered the main production and high-yield region of wheat and potato in Guizhou Province. The sampling locations for wheat grains are shown in Figure 1.

In 2021, a total of 149 wheat grain samples at the mature period were collected by the regular systematic composite grid sampling technique, and each representative wheat grain sample was composed of 5 subsamples and then transported to the laboratory. The specific information on wheat grain species is shown in Supplementary Table 1. The longitude and latitude of each sampling site were recorded using the Global Positioning System (GPS). During collection, conservation, and pretreatment, wheat grain samples were prevented from touching other materials to refrain from pollution. After oven-dried at 72 h for 60°C to hold constant weight, the samples were stored and sealed in polyethylene bags until digestion.

### Chemical analysis

To determine the concentrations of Cr, Ni, Pb, Cd, and As, approximately 2 g of the stored wheat grain samples was digested with 20 ml of HNO_3_: HClO_4_ (5:1), and then heated in the draught cupboard (110–130°C) until a clear solution was gathered at about 1–2 h (24). The digested samples were filtered through a 0.45 μm filter (36). Thereafter, the concentrations of Cr, Ni, Pb, Cd, and As were measured by the ICP-MS (NexION 2000 ICP-MS, PerkinElmer, United States). Meanwhile, the determination of F concentration was conducted based on the Chinese Standard Method (GB/T5009.18 2003) (37). For the total F measurement in wheat grains, approximately 0.2 g of samples were digested with 2 ml of nitric acid (15 mol/L), 3 ml of hydrogen peroxide (10 mol/L), and 5 ml of ultrapure water, eventually measured with the fluoride-ion electrode method (Mettler-Toledo Instruments, Shanghai) (38).

### Quality control and quality assurance

In the process of wheat grain samples’ quality control in the laboratory, guarantee reagents (GRs) and all chemicals were used. The validation of the analytical program was conducted using subsequent standards such as blank reagents, calibration of the instruments, precision, and detection limit. Related experiment supplies (crucibles, glassware, and plastic containers) were washed with deionized water and then soaked in a 10% HNO_3_ solution for 24 h. In addition, they were rewashed with deionized water and oven-dried at 140–160°C for 24 h before use (39). Moreover, the samples were measured in duplicate for the quality assurance and the recoveries of PTE ranged from 85 to 90%.

### Non-carcinogenic risk assessment

There is a minimum dose (threshold values) to cause harm to human health for toxic and harmful substances exposure. If the minimum dose of human ingestion is less than the threshold, it indicates no health risks to human health posed by toxic and harmful substances. The HQ and hazard index (HI) are the estimated approaches of non-CR due to PTE exposure (40). If HQ and HI values are greater than 1, it indicates a significant health risk to humans, while lower than 1 is considered safe. The non-CR caused by PTE (Cr, Ni, As, Cd, Pb, and F) in this study is evaluated in the following formulas (8):


(1) $$H\,Q=\frac{C\times E\,F\times I\,R\times E\,D}{B\,W\times A\,T\times R_{f}\,D}$$

(2) $$H\,I=\sum_{i=1}^{n}H\,Q$$

where C indicates PTE concentration in wheat grains, mg/kg; EF indicates exposure frequency, day/year (365 for children, adult females, and adult males); ED indicates ED, year (6 for children and 70 for adult females and males); IR indicates the daily intake rate of wheat grains, mg/kg/day (0.094 for children and 0.160 for adult females and males); BW indicates body weight, kg (16.88 for children, 57.03 for adult females, and 66.2 for adult males); AT indicates the averaging time, year (ED × 365 for non-carcinogenic and 70 × 365 for carcinogenic) (41) (USEPA 2019); and R_*f*_D is the oral reference dose, mg/kg/day (F: 0.06; Cd: 0.001; Pb: 0.0035; As: 0.0003; Zn: 0.3; Ni: 0.02) (6) (USEPA 2019).

### Carcinogenic risk assessment

Carcinogenic risk indicates the probability of an individual suffering from cancer over a lifetime due to PTE exposure. CR is calculated using PTE intake and toxicity values (slope factors), and threshold CR (TCR) is the summation of CR (USEPA 1999). In this study, the CR is calculated as follows (40):


(3) $$C\,R=\frac{C\times E\,F\times I\,R\times E\,D}{B\,W\times A\,T}\times S\,F$$

(4) $$T\,C\,R=\sum_{i=1}^{n}C\,R$$

where SF is the slope factor of Cd, As, Pb, and Cr with 0.38, 1.5, 0.0085, and 0.5 (mg/kg/day), respectively (6). When CR (TCR) < 1 × 10^–6^, the CR can be ignored; when CR (TCR) ranges from 1 × 10^–6^ to 1 × 10^–4^, a cautionary risk cannot be negligible; when *CR* (*TCR*) > 1 × 10^–4^, there is an unacceptable CR, and relevant measures must be implemented to decrease the risk.

### Monte Carlo simulation and sensitivity analysis

In this study, the probabilistic risk assessment was performed by Monte Carlo simulation considering the distribution fitting and random sampling, and then the simulated results of the best output can be applied for uncertainty and sensitivity assessment (42). The benefit of the model is that the error of the method is independent of the dimension of the problem, and the distribution of human health risk can be simulated effectively (31). In addition, to identify the most influenced variables, the sensitivity analysis was also performed by employing a sensitivity coefficient. Using the Crystal Ball software, 10,000 simulation experiments in thus study were conducted using the important exposure parameters.

### Statistical analysis

All statistical analysis and figures were performed using SPSS version 22, Origin 2021pro, and ArcGIS 10.6 for Windows. Spearman’s correlation analysis was used to identify the correlations between investigated PTEs, which provided preliminary information for explaining the sources of PTE in the study region. Principal component analysis (PCA) was used to further explore the pollution sources of PTE in the study environment, and the validity was verified based on the Kaiser–Meyer–Olkin (KMO) value (> 0.5) and Bartlett sphericity tests (*p* < 0.001).

## Results

### Potentially toxic elements concentrations of wheat grains

**FIGURE 2:**
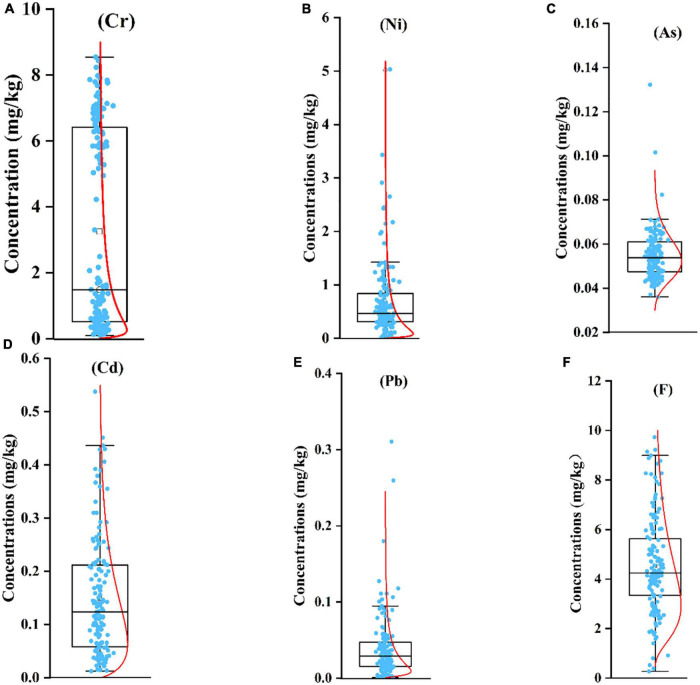
The PTE concentration (mg/kg) in wheat grain samples collected from Bijie, Guizhou Province [**(A)** Cr, **(B)** Ni, **(C)** As, **(D)** Cd, **(E)** Pb, **(F)** F]. The red line indicates “normal curve.”

The statistical summaries of PTE concentrations in the study area are presented in Table 1 and Figure 2. Mean concentrations of PTE in wheat grains decreased in the order: *F*(4.539 mg/kg) > Cr (3.250 mg/kg) > Ni (0.684 mg/kg) > Cd (0.149 mg/kg) > As (0.055 mg/kg) > Pb (0.039 mg/kg). Specifically, mean concentrations of As, Ni, Cd, and Pb all met the standard limits [Food and Agriculture Organization (FAO) 2020 (43)], while the mean Cr concentration was higher 3.25 times than 1 of the standard limit. Meanwhile, of the149 wheat grain samples, 87 for Cr, 30 for Cd, 43 for Ni, and 2 for Pb samples exceeded their respective standard limits. Therefore, the exceeding ratios of Cr, Cd, Ni, and Pb in wheat grain samples were 58.389, 28.859, 20.134, and 1.342%, respectively. Notably, although the related standard limit for F has been canceled, it is still critical to draw attention to its management, which the maximum value in our study had up to 9.730 mg/kg.

**TABLE 1:** The descriptive statistics of PTE concentrations in wheat grains (mg/kg).

Wheat	Cr	Ni	As	Cd	Pb	F
Mean	3.250	0.684	0.055	0.149	0.039	4.539
Median	1.488	0.468	0.054	0.124	0.029	4.243
SD	2.987	0.690	0.011	0.110	0.041	1.957
Minimum	0.096	0.000	0.036	0.012	0.001	0.272
Maximum	8.546	5.034	0.132	0.538	0.311	9.730
Standard limit	1.000	1.000	0.500	0.200	0.200	–
Exceeding ratio (%)	58.389	20.134	0.000	28.859	1.342	–
CV (%)	91.973	100.857	20.369	74.249	105.283	43.277

The coefficient value (CV) of variation was applied to evaluate the degree of discrete distribution of the concentrations of the PTE in wheat grains (44). CV < 10% indicated weak variation, which means that the concentration of PTE was mainly affected by natural factors; 10–90% indicated moderate variation; CV > 90% indicated strong variation, which means that the concentration of PTE was mainly impacted by anthropogenic activities (45). In this study, it showed a basic tendency of Pb (105.283%) > Ni (100.857%) > Cr (91.973%) > Cd (74.249%) > F (43.277%) > As (20.369%) (Table 1). These results showed that the Pb, Ni, and Cd in wheat grain showed massive variations depending upon the human factors in the study region.

### Source identification

To effectively reveal the natural and anthropogenic sources of PTE, the Spearman correlation analysis was used to initially identify the correlations between the concentrations of study PTE. The results (Table 2) showed that the Cr had a significantly positive correlation with Ni (*r* = 0.309, *p* < 0.01), As (*r* = −0.222, *p* < 0.01), and Pb (*r* = 0.472, *p* < 0.01). In addition, the positive correlations were observed in the pairwise comparisons of some PTE concentrations (Ni-Cd, Pb-Cd, and Ni-Pb) (*p* < 0.01). However, there were no statistical correlations between F and other PTEs.

**TABLE 2:** Correlation analysis for PTE in wheat grains of study area.

	Cr	Ni	As	Cd	Pb	F
Cr	1					
Ni	0.309**	1				
As	−0.222**	0.107	1			
Cd	–0.023	0.143*	0.120	1		
Pb	0.472**	0.228**	–0.191	0.079**	1	
F	0.044	–0.032	–0.081	0.006	0.045	1

The * and ** revealed that there were statistical significance. The * presented at the 0.05 level, and the ** presented at 0.01 level.

**FIGURE 3:**
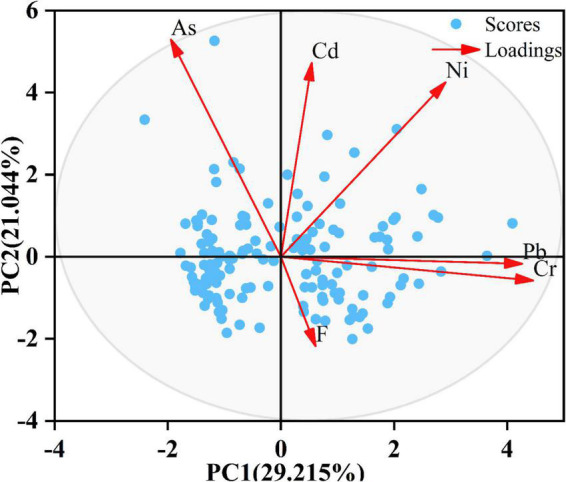
The PCA biplot showing the relationships of PTE concentrations in wheat grains.

Principal component analysis was further applied to identify PTE sources in wheat grains. The results of the KMO (0.652 > 0.5) and Bartlett spherical tests (0.000 < 0.05) show that the data were suitable for PCA. It demonstrates that the two principal components (PC1 and PC2) reflected 50.259% of variance information (Figure 3). PC1 was explained for 29.215% of the total variance and characterized by high loading factors of Cr (0.826) and Pb (0.790), and PC2 was dominated by Ni, As, and Cd accounting for 21.044% of the total variance, which indicates that the wheat grains contained high concentrations for them.

### Probabilistic risk

#### Non-carcinogenic risk

**FIGURE 4:**
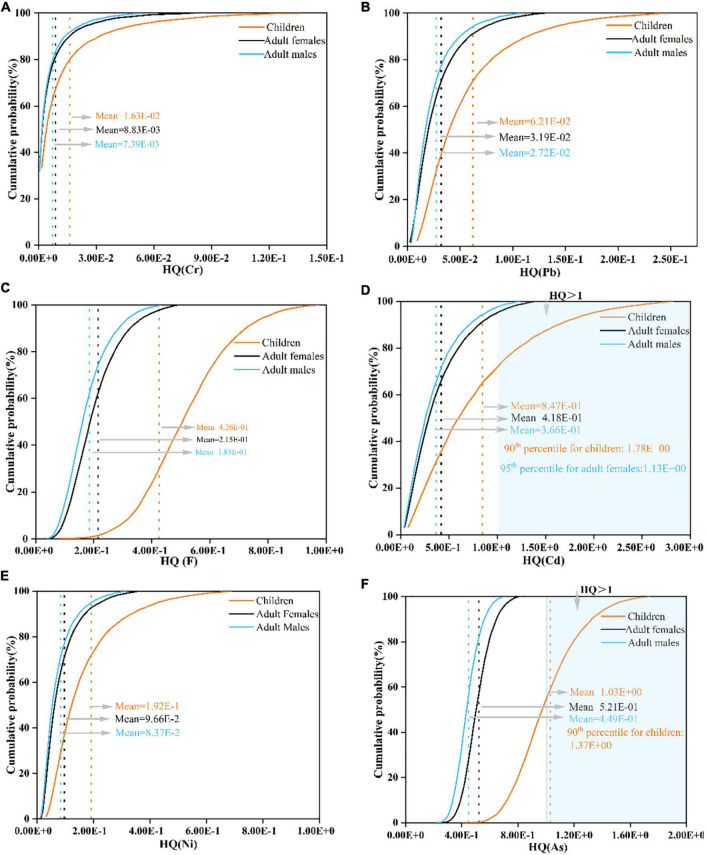
Cumulative probabilistic distributions of hazard quotient (HQ) for PTE through wheat grain consumption in three populations (children, adult females, and adult males). [**(A)** Cr; **(B)** Pb; **(C)** F; **(D)** Cd; **(E)** Ni; **(F)** As].

**FIGURE 5:**
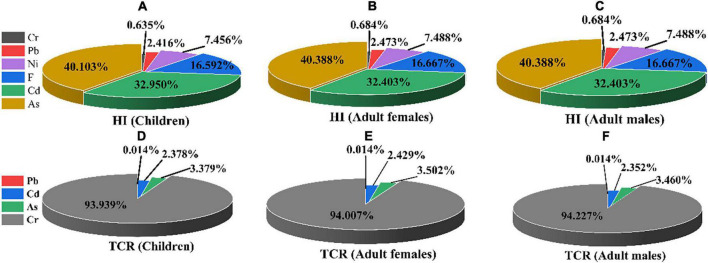
The contribution of hazard index (HI) and threshold carcinogenic risk (TCR) for children, adult females, and adult males. [**(A–C)** for HI; **(D–F)** for TCR].

The probabilistic non-CR of PTE in wheat grains, in terms of HQ and HI, was evaluated for children, adult females, and adult males (Table 3 and Figure 4). The mean values of simulated HQ for three populations all followed the order: As > Cd > F > Ni > Pb > Cr, which were all within the safe limit (1) except for As (children: 1.03E+00). It is worth noting that mean HQ values of As in all populations not only showed the largest level but were also the most contributor to HI (40.103% for children, 40.388% for adult females, and 40.388% for adult males) (Figure 5). In addition, the HQ values (90th percentile for children and 95th percentile for adult females in Cd, and 90th percentile for children in As) were higher than 1 and were 1.78E+00, 1.13E+00, and 1.37E+00, respectively, suggesting that there was a notable non-CR for exposure to Cd and As.

**TABLE 3:** Probabilistic evaluations of non-carcinogenic and carcinogenic risks for the PTE in wheat grains.

Risk	Element	Mean (median)			*SD*			95th percentile		
				
		Children	Adult females	Adult males	Children	Adult females	Adult males	Children	Adult females	Adult males
HQ	Cr	1.63E-02 (5.59E-03)	8.83E-03 (2.78E-03)	7.39E-03 (2.45E-03)	3.80E-02	2.62E-02	1.75E-02	6.31E-02	3.36E-02	2.88E-02
	Ni	1.92E-01 (1.33E-01)	9.66E-02 (6.71E-02)	8.37E-02 (5.83E-02)	1.94E-01	9.60E-02	8.41E-02	5.17E-01	2.61E-01	2.25E-01
	As	1.03E+00 (9.96E-01)	5.21E-01 (5.09E-01)	4.49E-01 (4.40E-01)	2.61E-01	1.05E-01	9.05E-02	1.51E-00	7.09E-01	6.14E-01
	Pb	6.21E-02 (4.23E-02)	3.19E-02 (2.14E-02)	2.72E-02 (1.84E-02	6.74E-02	3.54E-02	2.98E-02	1.75E-01	9.10E-02	7.79E-02
	Cd	8.47E-01 (6.42E-01)	4.18E-01 (3.11E-01)	3.66E-01 (2.75E-01)	7.15E-01	3.60E-01	3.15E-01	2.26E+00	1.13E-00	9.84E-01
	F	4.26E-01 (3.83E-01)	2.15E-01 (1.94E-01)	1.85E-01 (1.67E-01)	2.06E-01	1.01E-01	8.87E-02	8.20E-01	4.09E-01	3.52E-01
HI	Total	2.57E+00 (2.20E+00)	1.29E+00 (1.11E+00)	1.12E+00 (9.61E+01)	1.48E+00	7.23E-01	6.25E-01	5.35E+00	2.63E+00	2.28E+00
CR	Cr	1.24E-02 (4.23E-03)	6.21E-03 (2.07E-03)	5.47E-03 (1.78E-03)	3.39E-02	1.56E-02	1.63E-02	4.67E-02	2.38E-02	2.09E-02
	As	4.64E-04 (4.55E-04)	2.34E-04 (2.29E-04)	2.01E-04 (1.97E-04)	2.06E-06	4.74E-05	4.06E-05	6.32E-04	3.20E-04	2.75E-04
	Pb	1.89E-06 (1.26E-06)	9.32E-07 (6.34E-07)	8.17E-07 (2.44E-07)	2.65E-04	9.65E-07	2.65E-06	5.48E-06	2.60E-06	2.97E-06
	Cd	3.14E-04 (2.35E-04)	1.61E-04 (1.22E-04)	1.37E-04 (1.04E-04)	9.41E-05	1.36E-04	1.16E-04	8.42E-04	4.26E-04	3.70E-04
TCR	Total	1.32E-02	6.61E-03	5.81E-03	3.42E-02	1.57E-02	1.64E-02	4.82E-02	2.46E-02	2.16E-02

Moreover, compared to adults, children suffered from a greater probabilistic non-CR, and mean values of HI decreased in the following order: children (2.57E+00) > adult females (1.29E+00) > adult males (1.12E+00). Therefore, the non-CR assessment confirmed that adverse effect was observed for local citizens, especially in children.

#### Carcinogenic risk

**FIGURE 6:**
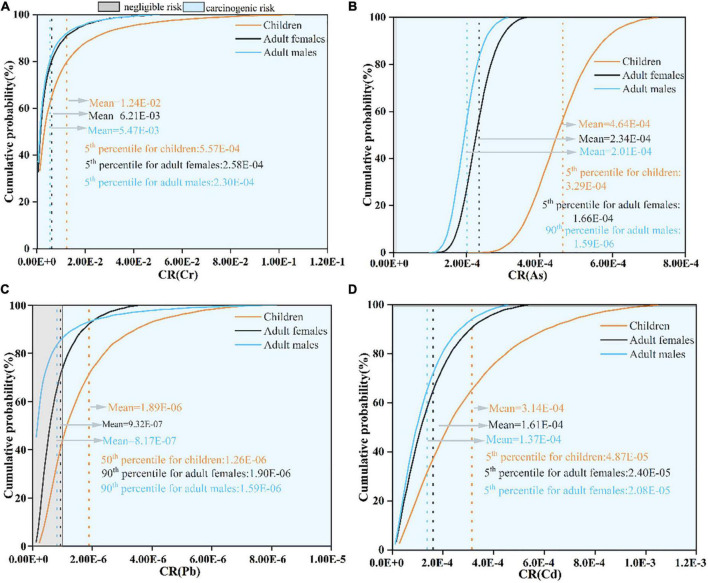
Cumulative probabilistic distributions of carcinogenic risk (CR) of PTE through wheat grain ingestion for three populations. [**(A)** Cr; **(B)** As; **(C)** Pb; **(D)** Cd].

In this study, only Cr, As, Pb, and Cd were considered CR. According to the probability distributions (Table 3 and Figure 6), the TCR values of four investigated PTEs cannot be negligible (>1E-06), while the CR values for individual PTE were different. The basic trend of mean CR values for each PTE in the three groups was all decreased in the order: Cr > As > Cd > Pb, which were all higher than the 1E-06 of the acceptable threshold except for Pb. Besides, the cumulative probabilities for both Cr and Cd exceeded the acceptable threshold (1E-06) in three populations (Figures 6A,D). More seriously, the CR values of Cr even at the 5th percentile were (557 times for children, 258 times for adult females, and 20.8 times for adult males) higher than the acceptable threshold (1E-06). These results showed that a cautionary CR existed in wheat grains for local citizens.

In addition, the total contribution to TCR in all populations was increased in the order of Pb < Cd < As < Cr, and more than 90% were attributed to Cr (Figure 5). Generally, the TCR values of children, adult females, and adult males were 1.32E-02, 6.61E-03, and 5.58E-03, respectively, which suggest that total CR could not be ignored and children were more susceptible than adults.

### Sensitivity analysis

**FIGURE 7:**
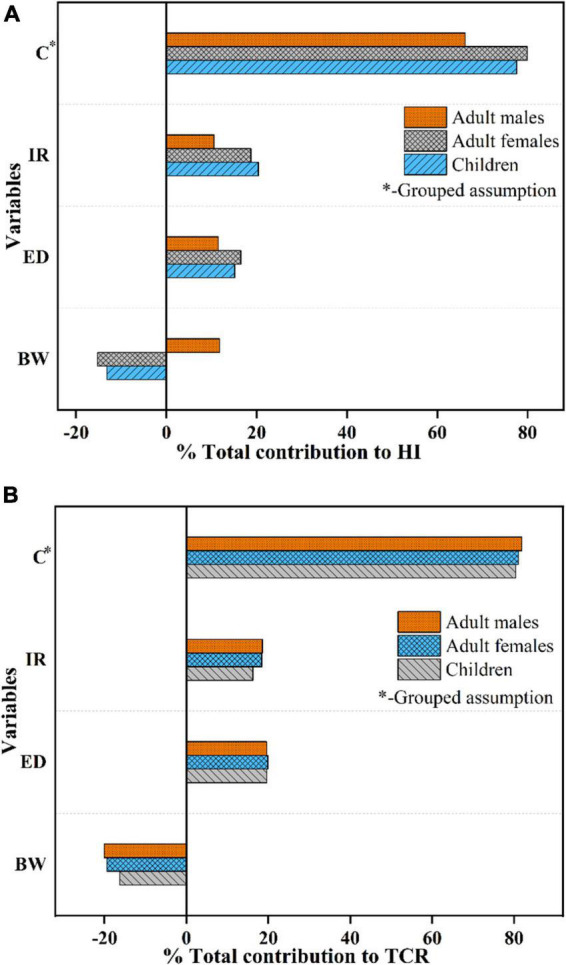
Sensitivity analysis of the input parameters for **(A)** hazard index (HI) and **(B)** threshold carcinogenic risk (TCR) for three populations (children, adult females, and adult males).

Sensitivity analysis for children, adult females, and adult males was applied to highlight the most crucial parameters contributing to the simulated results for probabilistic health risks (Figure 7). Our research implies that the concentration factor (C*) was the predominant parameter of the risk in all exposed populations, in which the highest contribution was up to 81.896%. Besides, the contributions for ED in three populations were nearly in line with IR, which ranged from 10.556 to 20.406% on HI and 16.242 to 19.892% on TCR, respectively. In contrast, inverse correlations were observed for BW on both TCR and HI (children and adult females). Overall, these differences might be attributed to the inaccuracy definitions of the probability distributions of ED, C*, IR, and BW.

## Discussion

### The distribution of potentially toxic elements concentration in wheat grains

Wheat is one of the most staple grown crops globally. In this study, the average values of Cd, As, Pb, and Ni in wheat grains were lower than the standard limits of FAO 2020 (43), while the Cr exceeded. Of note, the Cr concentrations were obviously distributed in two groups (Figure 2A), indicating uneven distribution was observed for its concentrations in wheat grains. Meanwhile, the same finding was also confirmed by the CV value of Cr (91.973%). It may be attributed to anthropogenic activities, including agrochemicals, sewage irrigation, and mineral mining. Ali et al. showed that the Cr concentrations in wheat grain irrigated by wastewater were beyond the standard limit and higher than that irrigated by uncontaminated water (22). Other studies approved that the high accumulation of PTE in wheat grain was due to their planting soils polluted with PTE (46, 47). Furthermore, Qin et al. showed that the enrichment capacities for Cd and As in the Xumai-30 specie were higher than that in the Baomai-2 specie (48). A field experiment suggested that the accumulating ability of PTE varied between different varieties as well (49). In view of the discussions earlier, PTE concentrations in the wheat grains were possibly related to the geographical origin and species.

Several studies have illustrated the PTE contamination status of wheat grains globally (50–52) (Table 4). The distributions of the same PTE significantly varied in different areas, even within the same region. The mean concentrations of Ni in this study were less than that reported in most of the studies (Table 4), while 14.766 times higher than that reported in Kurram river, Pakistan (22). Similarly, the mean As concentration was less than in other studies while higher than that reported in the United States and other countries (53). In contrast, the average Cd concentration was higher than that given in Table 4 with the exception of Niger Delta in Nigeria, Murcia in Spain, and Veles in Macedonia (54). Overall, the concentrations of PTE in wheat grains grown in the areas of contaminated water and mining were obviously higher than those in other regions. However, there were less studies focused on F and other PTEs. The mean F concentration in this study was higher than that reported by Li et al. (55) while lower than that reported by Jha et al. (56). Due to the difference in climate between the north and the south, there were differences in the planting and harvest periods between each reported region. Thus, the variations for PTE accumulating in wheat among regions both domestic and abroad (57, 58) may be greatly related to the differences in climate and harvest season. Meanwhile, in turn, the rainfall and temperature factors (pH, organic matter) could indirectly affect the bioavailability, to change the absorption of PTE in plants.

**TABLE 4:** Comparison of PTE concentrations in wheat grains from other studies (mg/kg).

County	n	PTE						Reference
		Cr	Ni	As	Pb	Cd	F	
Bijie, China	149	3.250	0.684	0.055	0.039	0.149	4.539	This study
Baoji, China	81	0.100	0.090	–	0.030	0.013	–	(21)
Kurram river, Pakistan	25	7.410	10.100	–	9.400	0.120	–	(22)
Konya, Turkey	9	4.000	1.600	0.900	1.300	0.100	–	(50)
The United States and other countries	28	–	–	0.008	0.010	0.011	–	(53)
Ladakh, India	70	–	–	0.110	0.070	0.050	–	(22)
Xidagou, China	20	0.500	0.900	–	0.090	0.090	0.300	(55)
Dongdagou, China	22	0.400	0.900	–	0.060	0.070	0.200	(55)
Bangladesh	30	0.352	0.145	0.281	0.221	0.011	–	(51)
Egypt	–	0.130	0.380	–	0.140	0.140	–	(52)
Uttar Pradesh, India	–	–	–	–	–	–	8.800	(56)
Niger Delta in Nigeria	–	0.910	–	3.300	0.530	0.170	–	(54)
Kenya	–	0.440	–	0.420	0.180	0.060	–	(54)
European Union Member States	–	1.010	–	2.550	0.410	0.020	–	(54)
Murcia in Spain	–	1.020	–	0.620	1.160	1.090	–	(54)
Veles in Macedonia	–	0.710	–	0.070	1.400	1.530	–	(54)
Faisalabad, Pakistan	96	–	0.715	–	0.339	–	–	(46)
Arequipa, Peru	16	–	–	–	2.235	0.114	–	(57)
Cape Verde archipelago	126	0.13	0.25	–	0.07	0.013	–	(58)

Potentially toxic elements could accumulate in plants through roots, straws, and grains. Previous studies have shown that the roots could represent PTE for soil decomposers, and the straws could be used as animal feed to impact animal health and ultimately human health *via* meat and milk (4). In addition, Tang et al. showed that the concentrations of Cd, Cr, As, and Pb in plant tissues were in the following order: root > straw > grain (59). It thus cannot be neglected that the PTE accumulates in roots and straws. More importantly, what caused the variations of PTE concentrations may be attributed to the distribution and excretion of PTE in local soil (pH, organic matter), as well as their bioavailability and plant’s metabolism (22). For example, the increased soil pH could reduce the Cd bioavailability and Cd content in plants (60). Therefore, comprehensive consideration of the relationship of the PTE concentration from soil to plant and the plant parts (roots, straws, and grains) was conducted to evaluate the PTE bioconcentration, which would help estimate health risks and take steps to ensure food safety.

### Source identification of potentially toxic elements in wheat grains

The combination of Spearman correlation and PCA analyses could provide more persuasive information for source identification. PC1 (Cr and Pb) was in line with the correlation results. The study area was an agricultural planting area with some industries, in which the common source of Cr and Pb might be preliminarily contributed by the prolonged sewage discharge to the local environment (61). However, correlation results showed that Ni and Cd and As and Cd were not strongly correlated, indicating that there might be mixed contamination sources between them. References showed that agricultural supplies, such as pesticides, fertilizers, and other agricultural applications, were widely used in the research region and contained high Cd levels, which were partly absorbed by crops (62). Besides, Jiang et al. (63) reported that Cd existed mainly in chemical fertilizers in their investigated fields. Therefore, human activities should be mainly considered the potential Cd sources, while Ni and As sources were slightly influenced by natural factors. These results showed that the related PTE shared a potential similar source or common feature in the transformation or common transport pathway under the same chemical and physical conditions at sampling locations (64).

Interestingly, the results of correlation and PCA analyses suggested that F had different sources from other PTEs. The study area was located in Southwestern Guizhou, which had been reported that it existed the high F characteristics formed in the weathering processes of rocks and various sediments (60). Besides, compared to mean F background values (478 mg/kg) of soils in China, the average F concentration (929 mg/kg) in Guizhou was nearly higher 2 times than the former. Herein, what causes the source differences between F and other PTEs could be put down to the special geological characteristics of Guizhou.

### Uncertainties and prospects of the probabilistic risk assessment

For the non-CR, As was the critical pollutant in our result. Studies also showed that the exposure to As in crops was the largest contributing factor to human health risk, indicating that closer attention should be paid to the ingestion of As (23, 65). From the CR assessment, the Cr was the most essential contributor to non-CR. Similarly, previous studies have also shown that there was a cautionary risk of Cr exposure to local citizens (23, 66). Herein, the prevention and control of CR implemented in this study area should be drawn attention to Cr exposure. Generally, children more easily suffered from the non-carcinogenic and CRs, the findings similar to previous studies (50, 67). In conclusion, persistent health effects will be profound for local citizens if urgent remedial steps are not carried out.

The Monte Carlo simulation technique was carried out based on the probabilistic distribution of the exposure parameters, such as C*, IR, ED, and BW (68). However, uncertainties of the model itself were still inevitable, which may induce some variations in results. First, despite the samples being collected and handled according to the relevant national standards, the concentrations of PTE still had some errors to some extent, such as the selection of the sampling sites, conservation, and laboratory analysis (30). Our findings also showed that the concentrations of PTE (C*) were the most prominent factor that mostly affected the whole risk outcome. Besides, the probability distributions of critical parameters (BW, IR, and ED) were mainly derived from other studies, which might not be adapted to the specific conditions of our study area. Meanwhile, there were some biases when the health risks were calculated for local persons due to the racial and geographical diversities, which the parameters (e.g., RfD, SF, and EF) were mainly referenced from the USEPA.

In addition, antagonistic and synergistic correlations were observed between PTEs, such as Cd-Pb, Cd-F, Cd-Cu, and F-As, in previous studies (69–72). Therefore, our results were possible to be overestimated or underestimated since the total risks were evaluated by the simple summation of a single PTE. More importantly, some researchers confirmed that the bioavailability of every PTE significantly varied for individual populations (29), which might also result in an overestimation of health risk calculated using the total PTE concentrations. Synthesizing all discussions earlier, to improve the reliability of health risk assessment, it is better to obtain the actual data of relative parameters (e.g., IR, BW, and EF) for the local populace and to comprehensively consider spatial analysis and deterministic and probabilistic techniques in future research. Despite the uncertainties and limitations, the results from this study may provide some valuable suggestions for environmental managers and scientists, to reduce PTE exposure and prevent adverse threats to local residents.

## Conclusion

As one of the most significant crops, special attention to wheat is required to ensure food security. The Cr exposure to wheat grains in Bijie carried more serious risk since its average concentration was much higher than the standard limit set by FAO 2020 (43). As for the source identification, we predict that Cr and Pb might mainly originate from industrial activities, while Ni, As, and Cd should be from mixed sources (human factors and natural activities) and F was possibly put down to high geological background value. Residents, especially children, in the study area suffered from the probabilistic non-carcinogenic and CRs, among the most contributors were As and Cr, respectively. Therefore, to reduce the health risk of PTE in the study region, corresponding urgent measures should be undertaken.

## Data availability statement

The original contributions presented in this study are included in the article/Supplementary material, further inquiries can be directed to the corresponding author.

## Author contributions

QZ constructed the project. DL and CZ carried out all experiments, analyzed and interpreted the data, and drafted the manuscript. XL, FL, SL, and YZ performed the material preparation, data collection, and analysis. ZW and DS assisted in conducting the search and data. All authors read and approved the final manuscript.

## References

[1] Baruah SG Ahmed I Das B Ingtipi B Boruah H Gupta SK Heavy metal(loid)s contamination and health risk assessment of soil-rice system in rural and peri-urban areas of lower brahmaputra valley, northeast India. *Chemosphere.* (2021) 266:129150. 10.1016/j.chemosphere.2020.129150 33310523

[2] Setia R Dhaliwal SS Singh R Kumar V Taneja S Kukal SS Phytoavailability and human risk assessment of heavy metals in soils and food crops around Sutlej river, India. *Chemosphere.* (2021) 263:128321. 10.1016/j.chemosphere.2020.128321 33297254

[3] Mng’ong’o M Munishi LK Ndakidemi PA Blake W Comber S Hutchinson TH. Toxic metals in East African agro-ecosystems: Key risks for sustainable food production. *J Environ Manag.* (2021) 294:112973. 10.1016/j.jenvman.2021.112973 34102465

[4] Mng’ong’o M Munishi LK Ndakidemi PA Blake W Comber S Hutchinson TH. Accumulation and bioconcentration of heavy metals in two phases from agricultural soil to plants in Usangu agroecosystem-Tanzania. *Heliyon.* (2021) 7:e07514. 10.1016/j.heliyon.2021.e07514 34296014 PMC8282977

[5] Kukusamude C Sricharoen P Limchoowong N Kongsri S. Heavy metals and probabilistic risk assessment *via* rice consumption in Thailand. *Food Chem.* (2021) 334:127402. 10.1016/j.foodchem.2020.127402 32711260

[6] Pipoyan D Stepanyan S Stepanyan S Beglaryan M Merendino N. Health risk assessment of potentially toxic trace and elements in vegetables grown under the impact of Kajaran mining complex. *Biol Trace Element Res.* (2019) 192:336–44. 10.1007/s12011-019-01675-w 30788723

[7] Zhao K Zhang L Dong J Wu J Ye Z Zhao W Risk assessment, spatial patterns and source apportionment of soil heavy metals in a typical Chinese hickory plantation region of southeastern China. *Geoderma.* (2020) 360:114011. 10.1016/j.geoderma.2019.114011

[8] Sanaei F Amin MM Alavijeh ZP Esfahani RA Sadeghi M Bandarrig NS Health risk assessment of potentially toxic elements intake *via* food crops consumption: Monte Carlo simulation-based probabilistic and heavy metal pollution index. *Environ Sci Pollut Res.* (2021) 28:1479–90. 10.1007/s11356-020-10450-7 32840749

[9] Jahandari A. Pollution status and human health risk assessments of selected heavy metals in urban dust of 16 cities in Iran. *Environ Sci Pollut Res.* (2020) 27:23094–107. 10.1007/s11356-020-08585-8 32329009

[10] Plum LM Rink L Haase H. The essential toxin: Impact of zinc on human health. *Int J Environ Res Public Health.* (2010) 7:1342–65. 10.3390/ijerph7041342 20617034 PMC2872358

[11] Anastasiou E Lorentz KO Stein GJ Mitchell PD. Prehistoric schistosomiasis parasite found in the Middle East. *Lancet Infect Dis.* (2014) 14:553–4. 10.1016/s1473-3099(14)70794-7 24953264

[12] World Health Organization [WHO]. *Fluorides Environmental Health Criteria 227.* Geneva: World Health Organization. (2002).

[13] Yu YQ Yang JY. Health risk assessment of fluorine in fertilizers from a fluorine contaminated region based on the oral bioaccessibility determined by Biomimetic Whole Digestion-Plasma *in-vitro* Method (BWDPM). *J Hazard Mater.* (2020) 383:121124. 10.1016/j.jhazmat.2019.121124 31505426

[14] Tattibayeva D Nebot C Miranda JM Abuova AB Baibatyrov TA Kizatova MZ A study on toxic and essential elements in wheat grain from the Republic of Kazakhstan. *Environ Sci Pollut Res.* (2016) 23:5527–37. 10.1007/s11356-015-5728-4 26573314

[15] Sharma S Nagpal AK Kaur I. Heavy metal contamination in soil, food crops and associated health risks for residents of Ropar wetland, Punjab, India and its environs. *Food Chem.* (2018) 255:15–22. 10.1016/j.foodchem.2018.02.037 29571461

[16] Ma G Jin Y Li Y Zhai F Kok FJ Jacobsen E Iron and zinc deficiencies in China: What is a feasible and cost-effective strategy? *Public Health Nutr.* (2008) 11:632–8. 10.1017/S1368980007001085 17894916

[17] Girolamo DE Cervellieri AS Cortese M Porricelli ACR Pascale M Longobardi F Fourier transform near-infrared and mid-infrared spectroscopy as efficient tools for rapid screening of deoxynivalenol contamination in wheat bran. *J Sci Food Agric.* (2019) 99:1946–53. 10.1002/jsfa.9392 30270446

[18] Ran J Wang D Wang C Zhang G Zhang H. Heavy metal contents, distribution, and prediction in a regional soilwheat system. *Sci Total Environ.* (2016) 544:422–31. 10.1016/j.scitotenv.2015.11.105 26657387

[19] Onipe OO Jideani AIO Beswa D. Composition and functionality of wheat bran and its application in some cereal food products. *Int J Food Sci Technol.* (2015) 50:2509–18. 10.1111/ijfs.12935

[20] Al-Othman ZA Ali R Al-Othman AM Ali J Habila MA. Assessment of toxic metals in wheat crops grown on selected soils, irrigated by different water sources. *Arabian J Chem.* (2016) 9:S1555–62. 10.1016/j.arabjc.2012.04.006

[21] Yang W Wang D Wang M Zhou F Huang J Xue M Heavy metals and associated health risk of wheat grain in a traditional cultivation area of Baoji, Shaanxi, China. *Environ Monit Assess.* (2019) 191:428. 10.1007/s10661-019-7534-9 31187274

[22] Ali J Khan S Khan A Waqas M Nasir MJ. Contamination of soil with potentially toxic metals and their bioaccumulation in wheat and associated health risk. *Environ Monit Assess.* (2020) 192:138. 10.1007/s10661-020-8096-6 31980942

[23] Yang S Zhao J Chang SX Collins C Xu J Liu X. Status assessment and probabilistic health risk modeling of metals accumulation in agriculture soils across China: A synthesis. *Environ Int.* (2019) 128:165–74. 10.1016/j.envint.2019.04.044 31055203

[24] Ihedioha JN Ogili EO Ekere NR Ezeofor CC. Risk assessment of heavy metal contamination of paddy soil and rice (Oryza sativa) from Abakaliki, Nigeria. *Environ Monit Assess.* (2019) 191:350. 10.1007/s10661-019-7491-3 31056719

[25] Peng J Li F Zhang J Chen Y Cao T Tong Z Comprehensive assessment of heavy metals pollution of farmland soil and crops in Jilin Province. *Environ Geochem Health.* (2019) 42:4369–83. 10.1007/s10653-019-00416-1 31535260

[26] Taiwo AM Oyeleye OF Majekodunmi BJ Anuobi VE Afolabi SA Idowu OE Evaluating the health risk of metals (Zn, Cr, Cd, Ni, Pb) in staple foods from Lagos and Ogun States, Southwestern Nigeria. *Environ Monit Assess.* (2019) 191:167. 10.1007/s10661-019-7307-5 30772926

[27] Hu B Jia X Hu J Xu D Xia F Li Y. Assessment of heavy metal pollution and health risks in the soil-plant-human system in the Yangtze River Delta, China. *Int J Environ Res Public Health.* (2017) 14:1042. 10.3390/ijerph14091042 28891954 PMC5615579

[28] Guo S Zhang Y Xiao J Zhang Q Ling J Chang B Assessment of heavy metal content, distribution, and sources in Nansi Lake sediments, China. *Environ Sci Pollut Res.* (2021) 28:30929–42. 10.1007/s11356-021-12729-9 33594554

[29] Sheng D Wen X Wu J Wu M Yu H Zhang C. Comprehensive probabilistic health risk assessment for exposure to arsenic and cadmium in groundwater. *Environ Manag.* (2021) 67:779–92. 10.1007/s00267-021-01431-8 33606066

[30] Jiang Y Ma J Ruan X Chen X. Compound health risk assessment of cumulative heavy metal exposure: A case study of a village near a battery factory in Henan Province, China. *Environ Sci Process Impacts.* (2020) 22:1408–22. 10.1039/d0em00104j 32458955

[31] Ramesh R Subramanian M Lakshmanan E Subramaniyan A Ganesan G. Human health risk assessment using Monte Carlo simulations for groundwater with uranium in southern India. *Ecotoxicol Environ Saf.* (2021) 226:112781. 10.1016/j.ecoenv.2021.112781 34563887

[32] Wen Y Li W Yang Z Zhang Q Ji J. Enrichment and source identification of Cd and other heavy metals in soils with high geochemical background in the karst region, Southwestern China. *Chemosphere.* (2020) 245:125620. 10.1016/j.chemosphere.2019.125620 31869671

[33] Gloaguen TV Passe JJ. Importance of lithology in defining natural background concentrations of Cr, Cu, Ni, Pb and Zn in sedimentary soils, northeastern Brazil. *Chemosphere.* (2017) 186:31–42. 10.1016/j.chemosphere.2017.07.134 28763636

[34] Meng W Shao-lin HE Chao-jin LI Wang F. Element geochemistry and fluoride enrichment mechanism in high-fluoride soils of endemic fluorosis -affected areas in Southwest China. *Earth Environ.* (2012) 40:2. in chinese. 10.14050/j.cnki.1672-9250.2012.02.001

[35] Wang M Zhang L Liu Y Chen D Liu L Li C Spatial variation and fractionation of fluoride in tobacco-planted soils and leaf fluoride concentration in tobacco in Bijie City, Southwest China. *Environ Sci Pollut Res.* (2021) 28:26112–23. 10.1007/s11356-020-11973-9 33483930

[36] Bhatti SS Kumar V Kumar A Kirby JK Gouzos J Correll R Potential carcinogenic and non-carcinogenic health hazards of metal(loid)s in food grains. *Environ Sci Pollut Res.* (2020) 27:17032–42. 10.1007/s11356-020-08238-w 32146668

[37] He L Tu C He S Long J Sun Y Sun Y Fluorine enrichment of vegetables and soil around an abandoned aluminium plant and its risk to human health. *Environ Geochem Health.* (2021) 43:1137–54. 10.1007/s10653-020-00568-5 32333231

[38] Rizzu M Tanda A Canu L Masawe K Mtei K Deroma MA Fluoride uptake and translocation in food crops grown in fluoride-rich soils. *J Sci Food Agric.* (2020) 100:5498–509. 10.1002/jsfa.10601 32567049

[39] Kaur L Rishi MS Siddiqui AU. Deterministic and probabilistic health risk assessment techniques to evaluate non-carcinogenic human health risk (NHHR) due to fluoride and nitrate in groundwater of Panipat, Haryana, India. *Environ Pollut.* (2020) 259:113711. 10.1016/j.envpol.2019.113711 31891909

[40] Hossain MB Ahmed ASS Sarker MSI. Human health risks of Hg, As, Mn, and Cr through consumption of fish, Ticto barb (Puntius ticto) from a tropical river, Bangladesh. *Environ Sci Pollut Res.* (2018) 25:31727–36. 10.1007/s11356-018-3158-9 30209769

[41] Peng Y Yang R Jin T Chen J Zhang J. Risk assessment for potentially toxic metal(loid)s in potatoes in the indigenous zinc smelting area of northwestern Guizhou Province, China. *Food Chem Toxicol.* (2018) 120:328–39. 10.1016/j.fct.2018.07.026 30016697

[42] Srinivasan R Rajasekhar B Nambi IM. Deterministic and probabilistic health risk assessment for exposure to non-steroidal anti-inflammatory drugs in an Indian river. *Environ Sci Pollut Res.* (2021) 28:39826–39. 10.1007/s11356-020-11897-4 33768453

[43] Food and Agriculture Organization [FAO]. *Food Standards Pro gramme Codex Committee on Contaminants in Foods.* (2020).

[44] Chen F Wang Q Meng F Chen M Wang B. Effects of long-term zinc smelting activities on the distribution and health risk of heavy metals in agricultural soils of Guizhou province, China. *Environ Geochem Health.* (2020): [Online ahead of print]. 10.1007/s10653-020-00716-x 32935252

[45] Huang T Deng Y Zhang X Wu D Wang X Huang S. Distribution, source identification, and health risk assessment of heavy metals in the soil-rice system of a farmland protection area in Hubei Province, Central China. *Environ Sci Pollut Res.* (2021) 28:68897–908. 10.1007/s11356-021-15213-6 34279778

[46] Abbas Q Yousaf B Liu G Zia-Ur-Rehman M Ali MU Munir MAM Evaluating the health risks of potentially toxic elements through wheat consumption in multi-industrial metropolis of Faisalabad, Pakistan. *Environ Sci Pollut Res.* (2017) 24:26646–57. 10.1007/s11356-017-0311-9 28956229

[47] Ahmad K Wajid K Khan ZI Ugulu I Memoona H Sana M Evaluation of potential toxic metals accumulation in wheat irrigated with wastewater. *Bull Environ Contamination Toxicol.* (2019) 102:822–8. 10.1007/s00128-019-02605-1 30955046

[48] Qin YH Qiang CK Zhang MH Cao JD Dan WF Han B. Difference analysis of accumulation and translocation of heavy metals in two wheat varieties and their health risk. *Mol Plant Breed.* (2019) 17:2742–8. (In Chinese). 10.13271/j.mpb.017.002742

[49] Qiang CK Qin YH Cao D Hu CX Zhang M Wang F Difference of heavy metal bioaccumulation among wheat varieties and their potential health risk assessment. *J Triticeae Crops.* (2017) 37:1489–96. (In chinese).

[50] Ozturk A Arici OK. Carcinogenic-potential ecological risk assessment of soils and wheat in the eastern region of Konya (Turkey). *Environ Sci Pollut Res.* (2021) 28:15471–84. 10.1007/s11356-020-11697-w 33237560

[51] Ahmed MK Shaheen N Islam MS Habibullah-Al-Mamun M Islam S Cp Banu CP. Trace elements in two staple cereals (rice and wheat) and associated health risk implications in Bangladesh. *Environ Monit Assess.* (2015) 187:326. 10.1007/s10661-015-4576-5 25944756

[52] Farahat EA Galal TM Elawa OE Hassan LM. Health risk assessment and growth characteristics of wheat and maize crops irrigated with contaminated wastewater. *Environ Monit Assess.* (2017) 189:535. 10.1007/s10661-017-6259-x 28971323

[53] TatahMentan M Nyachoti S Scott L Phan N Okwori FO Felemban N Toxic and essential elements in rice and other grains from the United States and other countries. *Int J Environ Res Public Health.* (2020) 17:8128. 10.3390/ijerph17218128 33153201 PMC7663342

[54] Wang S Wu W Liu F. Assessment of the human health risks of heavy metals in nine typical areas. *Environ Sci Pollut Res.* (2019) 26:12311–23. 10.1007/s11356-018-04076-z 30840254

[55] Li Y Wang S Nan Z Zang F Sun H Zhang Q Accumulation, fractionation and health risk assessment of fluoride and heavy metals in soil-crop systems in northwest China. *Sci Total Environ.* (2019) 663:307–14. 10.1016/j.scitotenv.2019.01.257 30711597

[56] Jha SK Nayak AK Sharma YK. Site specific toxicological risk from fluoride exposure through ingestion of vegetables and cereal crops in Unnao district, Uttar Pradesh, India. *Ecotoxicol Environ Saf.* (2011) 74:940–6. 10.1016/j.ecoenv.2011.01.002 21329982

[57] Rubio-Armendariz C Paz S Gutierrez AJ Gomes Furtado V Gonzalez-Weller D Revert C Toxic metals in cereals in cape verde: Risk assessment evaluation. *Int J Environ Res Public Health.* (2021) 18:3833. 10.3390/ijerph18073833 33917540 PMC8038792

[58] Choquenaira-Quispe C Angulo Vargas SJ Rojas-Tamata K Yucra Condori HR Villanueva Salas JA. Quantification and health risk assessment of lead and cadmium in wheat, rice, and their processed products from Peru. *J Environ Sci Health Part B.* (2022) 57:297–304. 10.1080/03601234.2022.2049152 35277121

[59] Tang L Deng S Tan D Long J Lei M. Heavy metal distribution, translocation, and human health risk assessment in the soil-rice system around Dongting Lake area, China. *Environ Sci Pollut Res.* (2019) 26:17655–65. 10.1007/s11356-019-05134-w 31028622

[60] Kong X Liu T Yu Z Chen Z Lei D Wang Z Heavy metal bioaccumulation in rice from a high geological background area in Guizhou Province, China. *Int J Environ Res Public Health.* (2018) 15:2281. 10.3390/ijerph15102281 30336616 PMC6211133

[61] Vatanpour N Feizy J Hedayati Talouki H Es’haghi Z Scesi L Malvandi AM. The high levels of heavy metal accumulation in cultivated rice from the Tajan river basin: Health and ecological risk assessment. *Chemosphere.* (2020) 245:125639. 10.1016/j.chemosphere.2019.125639 31864045

[62] Cai L Xu Z Ren M Guo Q Hu X Hu G Source identification of eight hazardous heavy metals in agricultural soils of Huizhou, Guangdong Province, China. *Ecotoxicol Environ Saf.* (2012) 78:2–8. 10.1016/j.ecoenv.2011.07.004 22257794

[63] Jiang F Ren B Hursthouse A Deng R Wang Z. Distribution, source identification, and ecological-health risks of potentially toxic elements (PTEs) in soil of thallium mine area (southwestern Guizhou, China). *Environ Sci Pollut Res.* (2019) 26:16556–67. 10.1007/s11356-019-04997-3 30982190

[64] Zhao Y Gao L Zha F Chen X Zhou X Wang X Research on heavy metal level and co-occurrence network in typical ecological fragile area. *J Environ Health Sci Eng.* (2021) 19:531–40. 10.1007/s40201-021-00625-w 34150256 PMC8172680

[65] Zhang Z Zhang N Li H Lu Y Yang Z. Potential health risk assessment for inhabitants posed by heavy metals in rice in Zijiang River basin, Hunan Province, China. *Environ Sci Pollut Res.* (2020) 27:24013–24. 10.1007/s11356-020-08568-9 32304056

[66] Zeng X Wang Z Wang J Guo J Chen X Zhuang J. Health risk assessment of heavy metals *via* dietary intake of wheat grown in Tianjin sewage irrigation area. *Ecotoxicology.* (2015) 24:2115–24. 10.1007/s10646-015-1547-0 26433741

[67] Wang N Han J Wei Y Li G Sun Y. Potential ecological risk and health risk assessment of heavy metals and metalloid in soil around Xunyang Mining Areas. *Sustainability.* (2019) 11:4828. 10.3390/su11184828

[68] Guo G Zhang D Wang Y. Probabilistic human health risk assessment of heavy metal intake *via* vegetable consumption around Pb/Zn Smelters in Southwest China. *Int J Environ Res Public Health.* (2019) 16:3267. 10.3390/ijerph16183267 31491979 PMC6765770

[69] Li X Li R Yan J Song Y Huo J Lan Z Co-exposure of cadmium and lead on bone health in a southwestern Chinese population aged 40-75 years. *J Appl Toxicol.* (2020) 40:352–62. 10.1002/jat.3908 31680290

[70] Husejnovic MS Jankovic S Nikolic D Antonijevic B. Human health risk assessment of lead, cadmium, and mercury co-exposure from agricultural soils in the Tuzla Canton (Bosnia and Herzegovina). *Arhiv Higijenu Rada Toksikologiju.* (2021) 72:268–79. 10.2478/aiht-2021-72-3533 34985839 PMC8785110

[71] Wang K Ma JY Li MY Qin YS Bao XC Wang CC Mechanisms of Cd and Cu induced toxicity in human gastric epithelial cells: Oxidative stress, cell cycle arrest and apoptosis. *Sci Total Environ.* (2021) 756:143951. 10.1016/j.scitotenv.2020.143951 33261865

[72] Chattopadhyay A. Environmental exposure of arsenic and fluoride and their combined toxicity: A recent update. *J Appl Toxicol.* (2020) 40:552–66. 10.1002/jat.3931 31867774

