# Supplementary material for: Co-exposure of potentially toxic elements in wheat grains reveals a probabilistic health risk in Southwestern Guizhou, China
Source: Front Nutr. 2022 Aug 8;9:934919. doi: 10.3389/fnut.2022.934919 (PMC9393542; doi:10.3389/fnut.2022.934919)
Supplement: Supplementary file 1 [file Table_1.docx]

Table S1. Specific information of wheat samples species collected from the study area

| Species | Qianmai-19 | Qianmai-20 |
| --- | --- | --- |
| Specie source | Guizhou Dry Grain Research Institute | Guizhou Dry Grain Research Institute |
| Validation number | Qianshen Mai 2011002 | Qianshen Mai 2014002 |
| Sowing period | From October 25th to November 5th | From October 25th to November 5th |
| Harvest period | Mid-May | Mid-May |
| Properties | Medium gluten wheat | Weak gluten wheat |
